# To Make Leeches Bite

**Published:** 1877-02

**Authors:** 


### TO MAKE LEECHES BITE.

The *Le Progres Medical* advises, in order to make a leech bite readily, to place it in a glass containing cold water, to wash the skin of the patient with warm water, and apply he glass containing the leech at once.

